# Neuronal expression of β2M and MHC I are essential for peripheral surveillance and targeting of neuron-restricted antigens

**DOI:** 10.3389/fneur.2026.1813496

**Published:** 2026-06-24

**Authors:** Benjamin D. S. Clarkson, Melanie E. Schille, Ivy J. Z. Garland, Maria Westphal, Charles L. Howe

**Affiliations:** 1 Department of Laboratory Medicine and Pathology, Mayo Clinic, Rochester, MN, United States; 2 Department of Neurology, Mayo Clinic, Rochester, MN, United States; 3 Center for Multiple Sclerosis and Autoimmune Neurology, Mayo Clinic, Rochester, MN, United States; 4 Division of Experimental Neurology, Mayo Clinic, Rochester, MN, United States

**Keywords:** β2M knockout, CD8^+^ T cell, cuprizone, EAE, MHC I, multiple sclerosis, OT-I, OVA

## Abstract

Neurons upregulate major histocompatibility complex class I (MHC I) during demyelination, enabling the presentation of self-antigens to cytotoxic CD8^+^ T cells. While this phenomenon is well described in multiple sclerosis lesions, its functional significance for disease progression remains poorly understood. Here, we test the hypothesis that neuronal MHC I expression promotes CD8^+^ T cell-mediated neurodegeneration in demyelinating disease. Using genetic and viral approaches to selectively ablate *β*₂-microglobulin (β₂M) in neurons, we demonstrate that the loss of neuronal MHC I limits antigen presentation, attenuates the activation of neuron-antigen–specific CD8^+^ T cells, and reduces the recruitment of these cells to the demyelinated central nervous system (CNS). In myelin oligodendrocyte glycoprotein (MOG₃₅–₅₅)-induced experimental autoimmune encephalomyelitis, CD8^+^ T cell ablation during active disease limited neuronal injury and improved clinical recovery. Similarly, neuronal *β*₂M ablation decreased clinical disease burden without affecting CNS T cell infiltration. Likewise, in the context of cuprizone intoxication, neuronal *β*₂M deletion reduced peripheral activation and CNS recruitment of CD8^+^ T cells recognizing a neuron-restricted neoantigen. It protected neoantigen-expressing neurons from cognate CD8^+^ T cell-mediated cytotoxicity. Collectively, these findings identify neuronal MHC I–dependent antigen presentation as a driver of both the immune surveillance of neuronal antigens and neuronal injury during demyelination.

## Introduction

Multiple sclerosis (MS) is a chronic autoimmune disease of the central nervous system (CNS) characterized by demyelination, neuroinflammation, and progressive neurological disability. For decades, the pathogenesis of MS has been understood primarily through the lens of myelin-reactive CD4^+^ T cell-mediated immunity, due to the prominent involvement of CD4^+^ T cells in experimental autoimmune encephalomyelitis (EAE) animal models and the strong genetic association with major histocompatibility complex (MHC) class II polymorphisms. This historical emphasis on CD4^+^ T cell immunity has obscured the role of CD8^+^ T cells and neuron-specific T cell responses, even though immunohistochemical studies have predominantly indicated that CD8^+^ T cells are the predominant lymphocytic infiltrate in MS lesions. Moreover, neuron loss and gray matter atrophy are the strongest correlates of disease progression. Yet, the extent to which these cytotoxic T cells drive irreversible neuronal loss in MS remains unclear.

Several reports have demonstrated that immunization with neuronal peptides or neuron-restricted antigens alone fails to induce clinical disease in mice, and that interferon-gamma (IFN-*γ*) induced neuronal MHC I upregulation is necessary for disease onset in these models (1–5). This is not surprising given the absence of sustained MHC I expression in healthy adult neurons (6, 7), even though the transient developmental expression of MHC I at synapses has been extensively described (8–11). The fact that adult neurons do not express MHC I molecules under homeostatic conditions represents a fundamental barrier to CD8^+^ T cell recognition of neuronal antigens in the steady-state conditions. However, adult neurons rapidly upregulate MHC I and the associated antigen-processing machinery in response to multiple pathogenic and inflammatory stimuli, including IFN-*γ* exposure (12, 13), the blockade of spontaneous electrical activity (14, 15), variable by viral infections (16–19), oxidative stress (4, 20), and critically, demyelination itself (21). Multiple independent studies have demonstrated that IFN-*γ* treatment of cultured mouse and human neurons drives the potent upregulation of MHC I, β2-microglobulin (β2M), and molecules involved in antigen processing (TAP, immunoproteasome subunits) on both neuronal cell bodies and axons. This IFN-*γ*-induced upregulation can occur through retrograde signaling from axons to neuronal cell bodies, resulting in anterograde transport of MHC I molecules into distal axons, where they are displayed on the axolemma (12). *In vivo* evidence similarly demonstrates that in demyelinating disease, neurons upregulate MHC I and β2M (21–24). Likewise, in human post-mortem brain tissue from patients with MS, we found elevated neuronal expression of MHC I transcripts and proteins specifically in the cortical and deep gray matter (21, 25).

This pathological upregulation of neuronal MHC I may therefore create a permissive microenvironment in which neuron-restricted antigens are presented on axonal and neuronal MHC I molecules, enabling antigen-specific CD8^+^ T cells to surveil, recognize, and directly injure antigen-presenting axons and neurons. In addition, while CNS meningeal lymphatics provide mechanisms of immune surveillance for sequestered CNS-specific antigens under normal physiological conditions (26–30), we have previously shown that demyelination enhances not only neuronal MHC I expression but also peripheral surveillance for neuron-restricted antigens, and that this second hit was necessary to drive the infiltration of neuron antigen-specific (NAS) T cells into the CNS (21). However, whether demyelination-induced neuronal MHC I upregulation was necessary for CD8^+^ T cell activation, CNS recruitment, or axonal injury has not yet been explicitly tested. Thus, we sought to determine the extent to which the surveillance and immunological targeting of neuron-restricted antigens are dependent on neuronal MHC I expression following demyelination and whether neuronal injury in the context of demyelination is dependent upon CD8^+^ T cell responses. Specifically, we tested whether ablating CD8^+^ T cells limits neuronal injury in EAE, whether ablating neuronal MHC I expression limits clinical disease and T cell recruitment to the CNS in EAE, and whether the ablation of neuronal surface MHC I expression limits peripheral activation and CNS recruitment of CD8^+^ NAS T cells and neuronal injury in the context of cuprizone-induced demyelinating disease.

## Materials and methods

### Study design and statistical methods

All statistical analyses were performed using GraphPad Prism (GraphPad Software). Data were assessed for distributional characteristics prior to hypothesis testing. Normality was assessed using the Shapiro–Wilk test when appropriate. Equality of variance was assessed prior to parametric testing; when variances were unequal, Welch’s correction was applied. For comparisons between two groups, normally distributed flow cytometric data were analyzed using unpaired two-tailed Student’s *t-*tests. In contrast, clinical scores (AUC) and immunostaining data were analyzed using the Mann–Whitney *U* test. For multiple comparisons, non-parametric (Kruskal–Wallis) tests were performed, followed by Dunn’s multiple-comparison test. Data are presented as the mean ± SEM unless otherwise indicated. Multiple comparison-adjusted *p-*values < 0.05 were considered statistically significant.

### Mice

C57BL/6(JAX# 000664), Thy1.1, (JAX# 000406), RFP (mT/mG, JAX #007676), β2M^fl/fl^ (JAX# 034858) Syn.Cre (JAX# 003966) and OT-I (JAX # 003831) were obtained from the Jackson Laboratory (Bar Harbor, ME). All F1 offspring used in experiments were screened for TCR-(Va2Vb5.1) and RFP or Thy1.1-transgene expression by flow cytometry on immune cells isolated from blood. Hemizygous Syn.Cre + β2M^fl/fl^ were generated by crossing Syn.Cre + mice with β2M^fll/fl^ mice, and backcrossing the resulting F1 generations with β2M^fl/fl^ mice. Mice were genotyped by gDNA isolation and PCR (Transnetyx). All animal experiments were approved by the Mayo Clinic Institutional Animal Care and Use Committee in accordance with National Institutes of Health guidelines.

### Cuprizone, EAE, and pertussis toxin injections

EAE was induced as previously described (31–33). Briefly, emulsion of equal volumes of CFA and 100 μg myelin oligodendrocyte glycoprotein peptide (MOG_35–55_, MEVGWYRSPFSRVVHLYRNGK) supplemented with *M. tuberculosis* H37Ra (5 mg/mL, Difco, Detroit, MI) was injected subcutaneously in the scapular region of each mouse. The MOG–CFA mixture was emulsified by sonication using a sonic dismembrator (Fisherbrand; FB705). Pertussis toxin (200 ng/mouse, i.p., List Biological Laboratories) was injected on days 0 and 2 relative to immunization. Clinical scores were monitored daily in a blinded manner and recorded as follows: 0, no clinical disease; 1, flaccid tail; 2, gait disturbance or hind limb weakness; 3, hind limb paralysis and no weight bearing on hind limbs; 4, hind limb paralysis with forelimb paresis and reduced ability to move around the cage; and 5, moribund or dead. Cuprizone (Bis(cyclohexanone)oxaldihydrazone; Sigma) diet containing 0.3% w/w cuprizone was prepared by Test Diet in the 5LG6 base diet and utilized within 6 months of manufacture. For experiments, mice were allowed access to experimental or control (5LG6) diet ad libitum and monitored weekly for 6 weeks prior to further manipulation.

### Intracerebral injections

For experiments involving cuprizone intoxication, 1 week prior to the cuprizone diet, adult mice were anesthetized and injected with 10 μL containing 2 × 10^9^ gene copies of AAV1.Syn.OVA-eGFP or AAV1.Syn.eGFP with or without co-injection of AAV1.Syn.Cre-mCherry 1.0 mm left of bregma at a depth of 1.25 mm below the surface of the brain using a 600 series 5ul Hamilton Syringe (No. 7633-01). The needle was held in place for 30 s following injection to prevent reflux upon needle removal. For experiments involving EAE, mice were injected at p0 (neonate) or 1 week prior to immunization (9–10 weeks of age). Adult mice were anesthetized and injected with 10 μL containing 2 × 10^9^ vector genomes (vg) of AAV1.Syn.Cre-eGFP or AAV1.Syn.eGFP-NLs 1.0 mm left and right of bregma at a depth of 1.25 mm below the surface of the brain using a Hamilton Syringe. The needle was held in place for 30 s following injection to prevent reflux upon needle removal. Neonatal mice were cryoanesthetized, and each hemisphere was injected with 1 μL containing 2×10^9^ gene copies of AAV1.Syn.Cre-eGFP or AAV1.Syn.eGFP-NLs 1 using a Hamilton Syringe. The needle was held in place for 30 s following injection to prevent reflux upon needle removal.

### Immunostaining and image analysis

For immunostaining, mice were perfused with > 15 mL of heparinized saline solution followed by > 30 mL of 4% paraformaldehyde (PFA) solution. Brains were collected and further post-fixed in 4% PFA for 24 h. Brains were then washed and transferred to a 30% sucrose solution prior to embedding. For floating sections, brains were embedded in 4% low-melting point agarose in PBS, and 70-micron floating sections were cut on a VT1000 P manual vibratome (Leica). Floating sections were permeabilized overnight in 0.1% Triton X-100 in TBS, then transferred to a blocking buffer containing 2.5% normal donkey serum, 5% bovine serum albumin, and 0.1% triton X-100 in TBS for 2 h. Floating sections were then transferred to blocking buffer containing primary antibodies (5 ug/mL), washed again, and counterstained with DAPI prior to mounting with VECTASHIELD Antifade Mounting Media (Vector Biolabs). For cryosections (8–20 micron), tissues were instead embedded in cryomolds with optimal cutting temperature (OCT) compound. Sections were cut at −18 °C to −20 °C on a cryostat, transferred to subbed slides, and immunostained as above. Images were acquired on a CKX41 microscope equipped with a CP73 camera (Olympus) using Cell View image acquisition software. Micrographs with excessive background or tissue folding were excluded from subsequent analyses. For semiquantitative analysis, digital images were processed and analyzed using Fiji (ImageJ) software as previously described (34). Briefly, following background subtraction, the polygon tool was used to select white matter tracts from the spinal cord for region-of-interest analysis. DAPI and A594 channels (MBP, SMI32, SMI312) were thresholded, and the positive staining area was measured relative to the DAPI area for each ROI. Average values (representative of 2–3 micrographs per mouse) are reported for each mouse. All contrast manipulations were applied equally to each image.

### Tissue processing for flow cytometry

For flow cytometric analysis, leukocytes were isolated from the brain, blood, deep cervical lymph nodes, or spleen as previously described (31, 33, 35). Briefly, blood (100 uL) was collected transcardially using an insulin syringe prior to perfusion with > 15 mL of heparinized saline solution. Blood was transferred to a 15 mL conical tube containing 10 mL Hank’s balanced salt solution with 10 mM EDTA and then centrifuged at 400 g for 5 min. Pellets were resuspended in HBSS, underlaid with 2 mL Histopaque-1077, and centrifuged at 600 g for 20 min to remove red blood cells. White blood cells were collected from the interface and upper layer, washed, and used for downstream analysis. Following perfusion, lymph nodes and spleen were gently dissociated in RPMI 1640 and passed through 40-micron cell strainers. Splenocytes were washed and resuspended in 1 mL of ACK containing RBC lysis buffer for 1 min and then washed again with > 10 mL of RPMI 1640 prior to counting 10^6^ lymphocytes or splenocytes were used from each mouse for each staining panel. Brains were minced with straight razors in RPMI 1640, then gently dissociated in tissue homogenizers and spun at 400 g for 5 min. Pellets were resuspended in 70% Percoll and 30% RPMI and overlaid with 30% Percoll 70% PBS prior to centrifugation at 2,200 g for 30 min with no brake. Leukocytes were collected from the interface, washed with > 10 volumes of RPMI, and split evenly across staining panels. For surface staining, single-cell suspensions were washed with FACS buffer containing 1% bovine serum albumin and 2 mM EDTA in PBS, then incubated for 30 min on ice in FACS buffer containing Fc Block (BD) or 25% v/v 2.4G2 cell supernatant and saturating concentrations of fluorophore-conjugated monoclonal antibodies. Cells were washed three times with FACS buffer and acquired on an Attune NxT (ThermoFisher) flow cytometer equipped with a 488-nm laser (filter set: 533/30, 585/40, 670LP) and a 640-nm laser (filter set: 675/25, 780/60).

### T cell transfers and labeling

For adoptive transfer, CD8^+^ T cells were purified from the lymph nodes of B6 or OT-I mice. Lymph nodes were gently dissociated, and cells were passed through a 40-micron cell strainer prior to magnetic isolation of CD8^+^ T cells with the Stem Cell Easy Sep kit. Briefly, single-cell suspensions were counted, brought up to 2 × 10^7^ cells per mL, labeled with an antibody cocktail for 30 min at room temperature, washed, and resuspended in 50 uL Streptavidin Plus rapid spheres per 10^7^ cells. After 15 min, cells were diluted and placed on the EasySep magnet for 6 min. Eluates were placed on a magnet for another 4 min, eluted once again, counted, and resuspended in 50% FBS/PBS at 10^7^ cells per mL for i.v. injection. Recipient mice received i.v. injection of 200 uL containing 2 × 10^6^ CD8^+^ T cells. CD8^+^ T cell purity was confirmed by immunostaining and flow cytometry.

## Results

### Ablation of CD8^+^ T cells at symptom onset reduces clinical disease burden and promotes motor recovery in MOG-immunization-induced demyelinating disease

**Figure 1:**
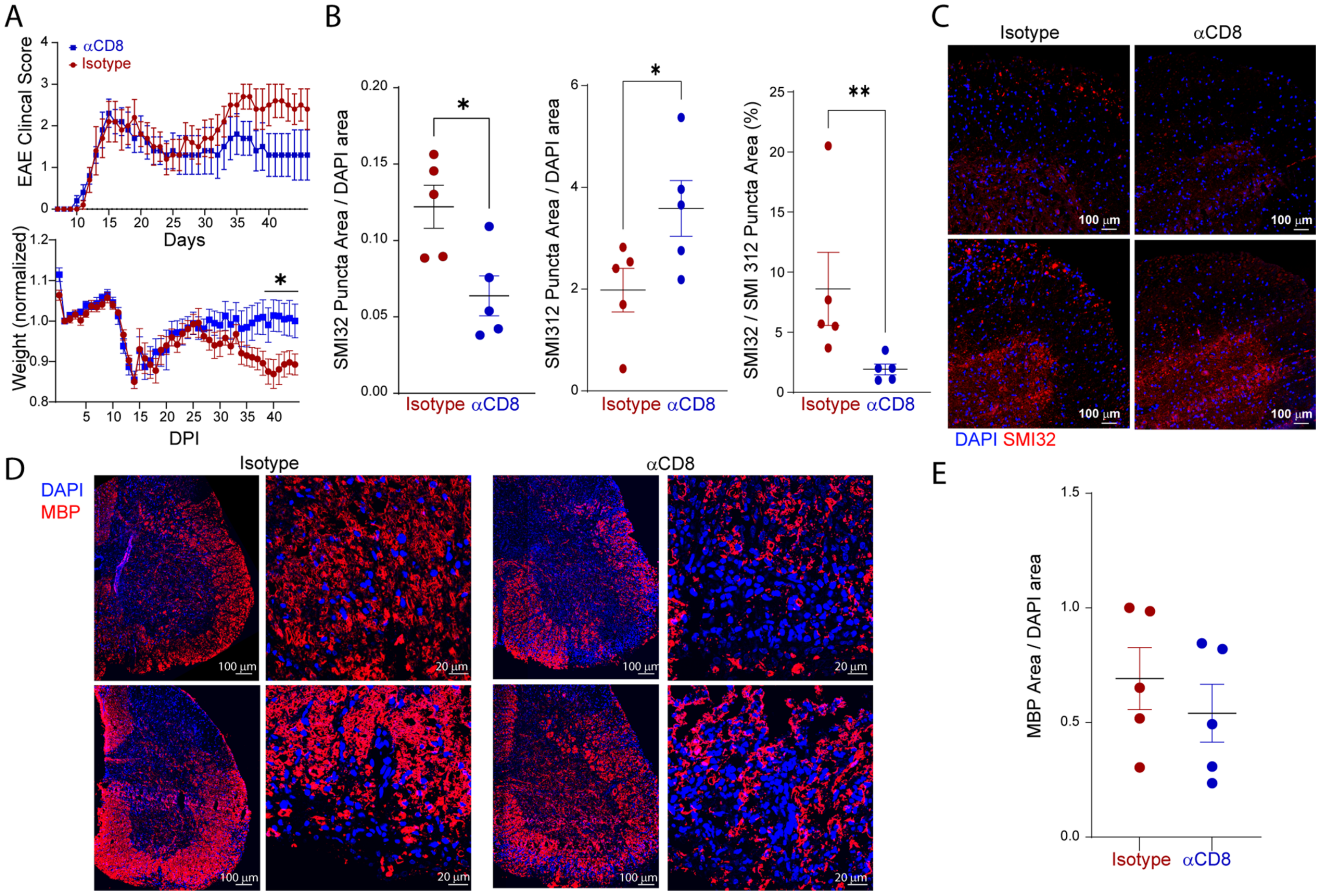
CD8 T cell depletion after clinical onset reduces axon injury and chronic worsening in EAE. C57Bl6 mice were immunized with MOG_35–55_ peptide to induce EAE. Mice received an IP injection of αCD8 or isotype control on days 18 and 21 post-immunization (DPI; *n* = 5 mice per group). **(A)** Clinical scores (0–5) and weight (normalized to 1 day post-immunization) are shown through 44 DPI. **(B)** Quantification of non-phosphorylated neurofilament (SMI32) vs. total neurofilament (SMI312) in sacral spinal cord of mice at end point, representative images shown in **(C)**. No differences in demyelination were observed between groups as assessed by MBP staining **(D)** as quantified in **(E)**. Error bars SEM. *p < 0.05; **p < 0.01.

We sought to determine the acute role of CD8^+^ T cells during the early effector phase of MOG₃₅_−_₅₅ EAE in C57BL/6 J mice. Mice were immunized with MOG₃₅_−_₅₅ peptide followed by pertussis toxin administration on days 0 and 2. To interrogate the specific contribution of CD8^+^ T cells during early disease progression, we administered depleting αCD8 monoclonal antibody (clone YTS169) or an isotype control (rat IgG2a) intraperitoneally on days 18 and 21 post-immunization, which rapidly depletes CD8 T cells from circulation and peripheral lymphoid organs (Supplementary Figures 1, 2) (36). This treatment window was deliberately selected to target CD8^+^ T cells during the transition from acute inflammatory infiltration to established CNS autoimmunity. At late time points, we observed substantial disease worsening in isotype control-treated mice that was not present in the majority of αCD8-treated mice (Figure 1A; clinical score area under the curve: 114.8 +/− 10.23 isotype control, 77.35 +/− 15.13 αCD8; Mean +/− SEM). Similarly, αCD8-treated mice exhibited significantly reduced weight loss at later time points compared with isotype controls. These findings are in contrast to prior studies demonstrating that αCD8 antibody depletion enhances EAE when given before disease induction (37)—instead, our findings suggest that ablating CD8 T cells during ongoing disease substantially ameliorates the disease when administered after symptom onset.

To determine whether CD8^+^ T cell depletion protected neurons and axons from injury in EAE independent of effects on demyelination, we performed quantitative immunohistochemical analysis on spinal cord cross-sections from αCD8- and isotype control-treated mice 10 weeks post-immunization. Specifically, in αCD8-treated mice, we observed a higher total axon count as well as a reduction in non-phosphorylated neurofilament H immunoreactivity (indicative of damaged or degenerating axons) in white matter tracts of the sacral spinal cord (Figures 1B,C). Critically, we observed no change in the overall extent of demyelination in spinal cord sections from αCD8-treated versus control mice, as assessed by MBP immunoreactivity (Figures 1D,E). Both groups exhibited similar areas of demyelinated white matter and similar spatial distributions of demyelination across spinal cord levels.

### Ablation of neuronal beta-2 microglobulin expression promotes recovery in MOG-immunization induced demyelinating disease

**Figure 2:**
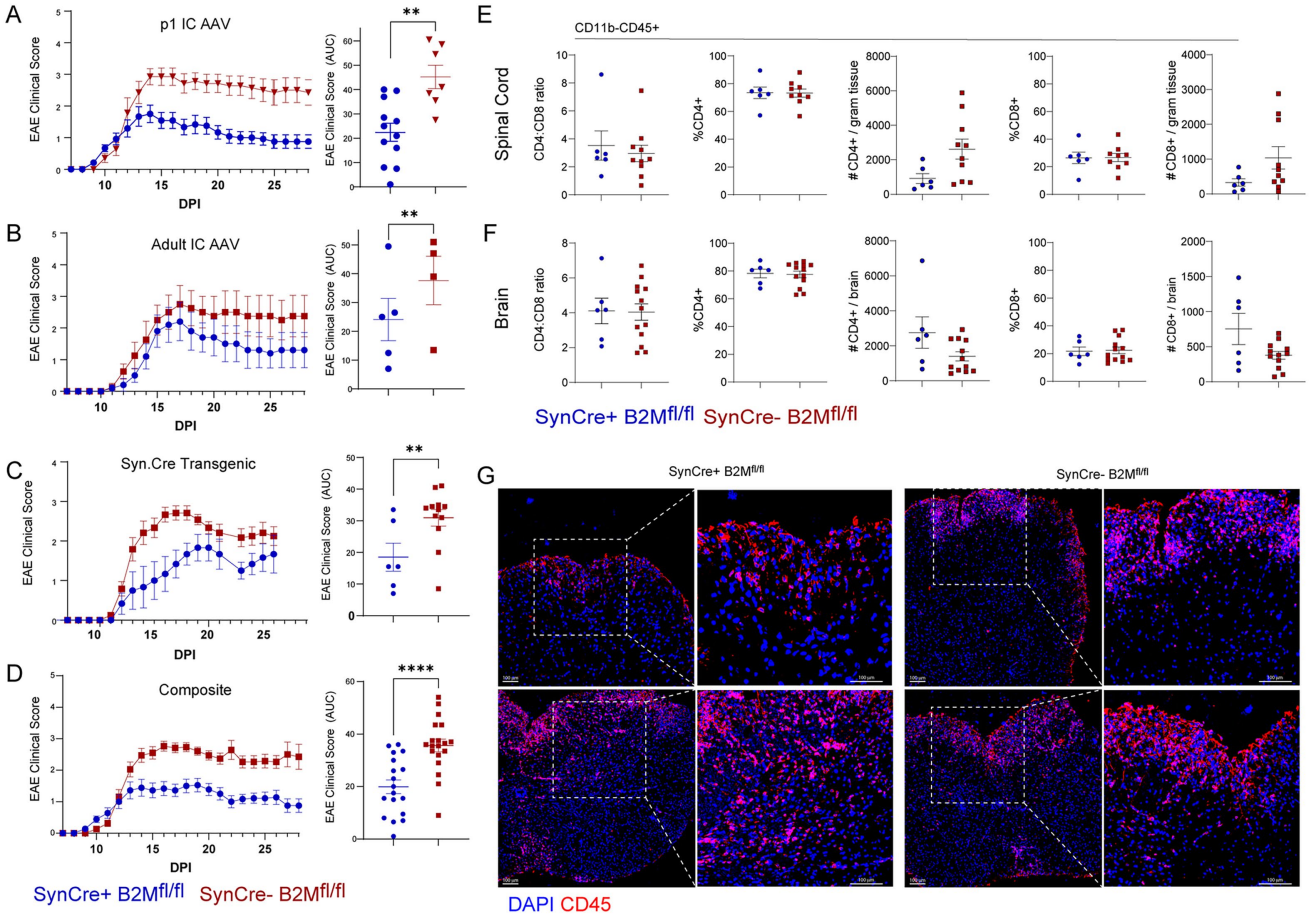
Ablation of neuronal β2M expression reduces clinical disease burden without impacting leukocyte recruitment. All mice were immunized with the MOG_35–55_ peptide to induce EAE. Prior to immunization β2M^fl/fl^ mice received i.c. injection of AAV1.Syn.Cre-EGFP [blue, **(A)**, *n* = 12; **(B)**, *n* = 5] or AAV1.Syn-EGFP control vector [red; **(A)**, *n* = 7; **(B)**, *n* = 4] at post-natal day 1 **(A)** or at 10 weeks of age **(B)**. **(C)** β2M^fl/fl^mice were crossed with Syn.Cre transgenic mice to generate Syn.Cre^+^β2M^fl/fl^ mice (blue; *n* = 6) or Syn.Cre^−^β2M^fl/fl^ littermate controls (red; *n* = 13). Composite scores across all conditions are shown in **(D)** (*n* = 23 Cre+, *n* = 24 Cre-). Daily scores (left) and area under the curve (AUC; right) are shown for each paradigm. Flow cytometric analysis of leukocytes infiltrating the spinal cord **(E)** and brain **(F)** is shown for Syn.Cre^+^β2M^fl/fl^ mice (blue) or Syn.Cre^−^β2M^fl/fl^ littermate controls from **(C)**. **(G)** Representative micrographs of CD45^+^ leukocytic infiltrate in the spinal cord at endpoint for mice from **(A)**. Error bars SEM. **p < 0.05, ****p < 0.0001.

We have previously demonstrated CD8^+^T cell-directed attack of neuron-restricted exogenous neoantigens following demyelinating disease (21). Therefore, we sought to determine whether CD8^+^ T cells target neuron-restricted endogenous antigens in the context of demyelinating disease. Since stable MHC I expression is dependent upon complex formation with β2M, we utilized conditional β2M knockout mice to achieve neuron-selective ablation of surface MHC I. In our first approach, we selectively ablated neuronal MHC I expression by injecting β2M^fl/fl^ neonates (P1) with AAV1.Syn.Cre-EGFP (neuronal β2M knockout) or AAV1.Syn.EGFP-NLS control vector. At 11 weeks, mice were immunized with MOG₃₅_−_₅₅ to induce EAE. Neuronal β2M ablation substantially reduced clinical disease severity, peak disease scores, and area under the curve for clinical scoring compared to control-injected littermates (Figure 2A). Because MHC I plays critical developmental roles in synaptic plasticity, synapse strengthening, and synaptic pruning during the postnatal period (11, 38–40), neonatal neuronal β2M ablation potentially confounds developmental effects with disease-relevant pathogenic functions. To distinguish these processes, we repeated the neuronal β2M ablation experiment using adult mice, injecting β2M^fl/fl^ mice with AAV1.Syn.Cre-EGFP or control vector 1 week prior to MOG₃₅_−_₅₅ immunization. Disease onset was comparable between groups, confirming that acute neuronal β2M ablation does not alter the initial induction of disease. However, neuronal β2M knockout demonstrated accelerated recovery from peak disease and reduced overall disease burden as assessed by area under the curve (Figure 2B)—effects similar to, though somewhat less pronounced than, those observed in neonatally injected mice. Histological confirmation of transgene expression revealed broader neuronal Cre-EGFP expression in P1-injected mice compared to adult-injected mice (Supplementary Figure 3), explaining the greater effect sizes in neonatally transduced animals despite both groups showing therapeutic benefit from neuronal MHC I ablation.

To confirm these findings with a constitutive transgenic model, we induced MOG₃₅_−_₅₅ EAE in B6-congenic Syn.Cre-β2M ^fl/fl^ mice and Cre-negative littermate controls. We have previously reported robust Cre expression in neurons across the neuraxis using Syn.Cre mice (21). Consistent with the AAV-mediated approach, Cre-positive mice exhibited delayed disease onset and substantially reduced overall clinical severity compared to controls (Figure 2C). Composite analysis of all EAE scores in all Syn-Cre + and Syn-Cre negative cohorts similarly demonstrated substantially reduced disease burden upon neuronal β2M ablation (Figure 2D). Surprisingly, despite a profound reduction of neuronal MHC I presentation, the proportional recruitment of CD8^+^ T cells to the CNS was comparable between groups, as assessed by both the CD8:CD4 ratio and percentage of CD11b^−^CD45^+^ lymphocytes (Figure 2E). Similarly, the proportion of brain and spinal cord-infiltrating lymphocytes that were CD4^+^ T cells was unaffected by neuronal β2M ablation (Figure 2E). Spinal cord tissue showed a non-significant trend toward reduced total CD4^+^ and CD8^+^ T lymphocytes in Cre-positive mice, an effect not observed in brain tissue (Figures 2E,F). Consistent with this, we found no qualitative differences in the pattern of CD45 + leukocyte infiltration into spinal cords (Figure 2G), suggesting that neuronal MHC I ablation reduces clinical disease severity through mechanisms independent of substantially altering overall T cell recruitment to the CNS.

### Neuronal β2M ablation limits neuron-antigen-specific CD8^+^ T cell activation and CNS recruitment

To directly measure the impact of neuronal β2M ablation on peripheral activation and CNS recruitment of CD8 + NAS T cells, we utilized our previously established model system for tracking NAS T cell responses (21). We injected β2M^fl/fl^ mice with AAV1.Syn.OVA-eGFP to drive neuron-specific expression of ovalbumin as a model neuron-restricted antigen, or with dual AAV vectors driving both OVA expression and Cre-mediated β2M deletion (AAV1.Syn.OVA-eGFP plus AAV1.Syn.Cre-RFP). Mice were then placed on a cuprizone diet to induce CNS demyelination, followed by adoptive transfer of purified CD8 + OT-I T cells (which specifically recognize OVA presented on MHC I), permitting real-time tracking of NAS T cell peripheral activation, proliferation, and CNS recruitment in the context of demyelination.

**Figure 3:**
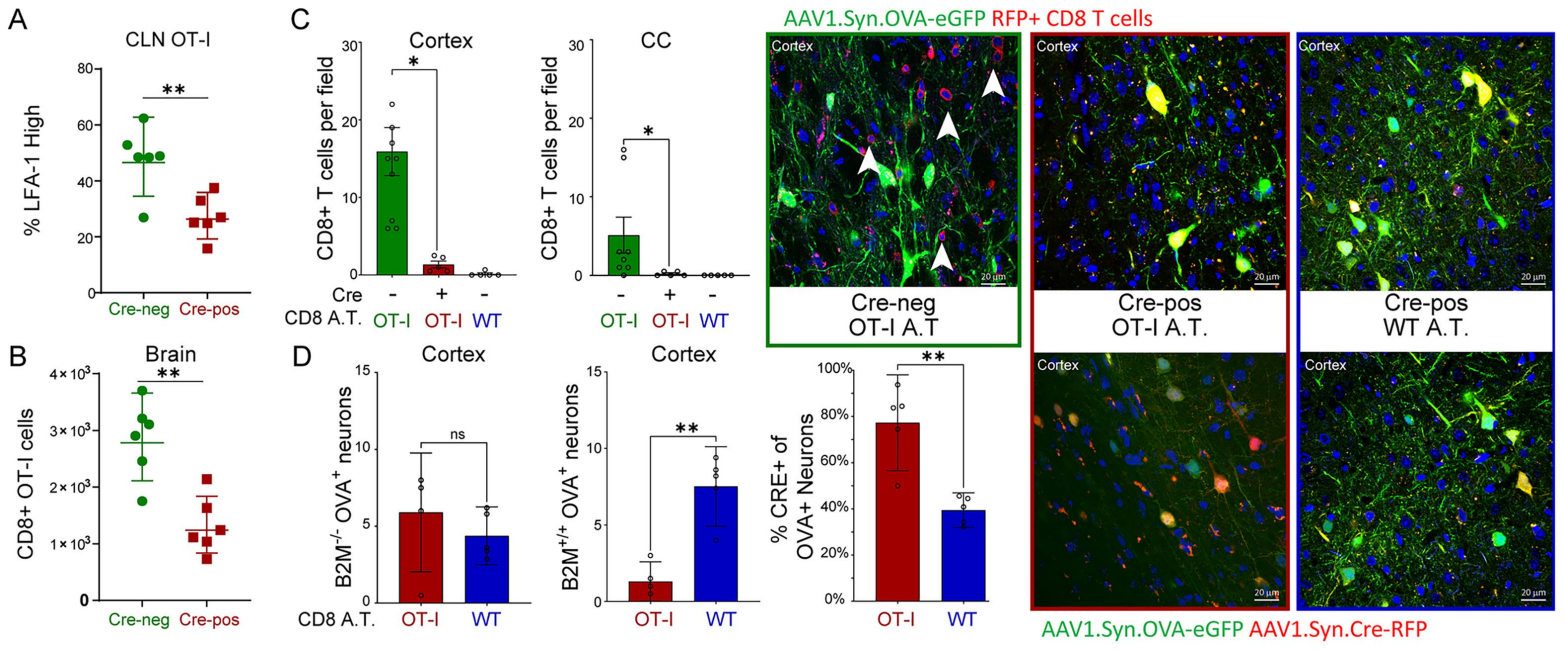
During cuprizone demyelination, ablation of neuronal β2M expression reduces peripheral activation and CNS recruitment of neural antigen-specific T cells. β2M^fl/fl^ mice were injected with AAV1.Syn.OVA-ATM-eGFP with (red) or without (green) coinjection of AAV1.Syn.Cre at 10 weeks of age. One week later, mice were initiated on a 0.3% cuprizone diet for 6 weeks to induce demyelination, after which they were adoptively transferred with 1×10^6^ CD8 + OVA-specific Thy1.1 OT-I T cells. Flow cytometric analysis 8 days after transfer of OT-I T cells showing **(A)** the percentage of CD8 + Thy1.1 + Va2 + Vb5.1 + OT-I T cells expressing high levels of activation marker LFA-1, and **(B)** absolute number of CD8 + Thy1.1 + Va2 + Vb5.1 + OT-I T cells recruited to the brain (*n* = 6 mice per group). **(C)** In separate experiments, β2M^fl/fl^ mice were injected with AAV1.Syn.OVA-ATM-eGFP with (red, blue; *n* = 5 each) or without (green; *n* = 9) coinjection of AAV1.Syn.Cre, initiated on a 0.3% cuprizone diet, and adoptively transferred with 1×10^6^ OT-I.RFP CD8^+^ T cells (green, red) or WT.RFP CD8^+^ T cells (blue) following the same paradigm. Brain sections were analyzed by confocal microscopy 8 days after adoptive transfer, and the average # CD8^+^ T cells enumerate in fields containing OVA-eGFP+ neurons. **(D)** β2M^fl/fl^ mice were injected with AAV1.Syn.OVA-ATM-eGFP and coinjected with reduced titers of AAV1.Syn.Cre-mCherry to generate mosaic expression of Cre^+^ OVA^+^ (β2M^−/-^OVA^+^) and Cre^−^OVA^+^(β2M^+/+^OVA^+^) neurons in cortex. Mice were initiated on a 0.3% cuprizone diet and adoptively transferred with 1×10^6^ OT-I CD8 + (red) or WT CD8^+^ T cells (blue) and OVA+ neuron subsets were analyzed by confocal microscopy 8 days later (*n* = 5 mice per group). Error bars SEM. *p < 0.05, **p < 0.01.

In brain-draining deep cervical lymph nodes, neuronal β2M ablation substantially reduced the expression of the T-cell activation marker LFA-1 on adoptively transferred NAS T cells (Figure 3A), indicating impaired peripheral priming and activation. Consistent with reduced peripheral activation, neuronal β2M ablation substantially reduced CNS accumulation of these cells, as measured by flow cytometric quantification in brain tissue (Figure 3B). To visualize CNS recruitment directly, in separate experiments, we adoptively transferred fluorescently labeled CD8 + OT-I T cells (RFP+) into cuprizone-demyelinated mice with either intact or ablated neuronal β2M expression. Consistent with the flow cytometric data, RFP + OT-I T cells accumulated to substantially lower levels in the brains of mice with neuronal β2M ablation compared to control mice (Figure 3C), demonstrating that loss of neuronal MHC I presentation reduces both peripheral priming and subsequent CNS recruitment of neuron-antigen-specific CD8^+^ T cells.

To determine whether neuronal β2M ablation protected neurons from direct NAS T cell-mediated cytotoxic injury, we employed a complementary experimental approach using selective neuronal deletion. β2M^fl/fl^ mice were co-transduced with AAV1.Syn.OVA-eGFP and lower titers of AAV1.Syn.Cre-RFP, generating a mosaic CNS in which approximately 30–40% of OVA-expressing neurons also expressed Cre recombinase and underwent β2M deletion (OVA^+^ β2M^−/−^ neurons), while the remaining OVA-expressing neurons retained normal β2M expression (OVA^+^β2M^+/+^ neurons). After 6 weeks on a cuprizone diet to induce demyelination, we adoptively transferred either wild-type CD8^+^ T cells or OT-I CD8^+^ T cells. OVA^+^ neuronal density was quantified 8 days post-transfer. As expected, wildtype CD8^+^ T cell transfer did not alter the density of either OVA^+^β2M^−/−^ or OVA^+^β2M^+/+^ neurons in contrast to OT-I CD8^+^ T cell transfer, which selectively reduced the density of OVA^+^β2M^+/+^ neurons but spared OVA^+^β2M^−/−^ neurons and resulted in >70% of remaining OVA+ neurons being Cre + (β2M−/−; Figure 3D). This selective sparing demonstrates that neuronal β2M ablation protects neurons from antigen-specific CD8^+^ T cell-mediated cytotoxic injury, providing direct evidence that MHC I-mediated antigen presentation is necessary for CD8^+^ T cell targeting of neuron-restricted antigens.

## Discussion

In animal models of MS, CD8^+^ T cells reactive to immunizing CNS antigens (e.g., immunodominant MOG_37-46_ or MBP) co-infiltrate the CNS along with CD4^+^ T cells, with evidence demonstrating that these cells are activated in brain-draining cervical lymph nodes, undergo clonal expansion within the CNS and peripheral tissues, and can be elicited to secrete pro-inflammatory cytokines in response to antigen stimulation (41–44). While some evidence suggests that CD8^+^ T cell recruitment to the CNS does not impact primary demyelinating disease outcomes in models of EAE (45), CNS-specific T cell populations, including MOG-specific and MBP-specific CD8^+^ T cells, have been shown to be capable of inducing severe CNS autoimmunity (43, 44). Indeed, MHC I-restricted myelin epitopes have been shown to be presented by oligodendrocyte lineage cells and cross-presented by infiltrating monocyte-derived dendritic cells (26, 31, 46, 47). Notably, CD8^+^ T cell-mediated EAE induced by the adoptive transfer of MOG-specific CD8^+^ T cells produces more severe, progressive, and destructive CNS lesions compared to disease actively induced by immunization, suggesting an enhanced tissue-damaging capacity of these cells (43). However, while these studies have almost exclusively focused on CD8^+^ T cell responses to classical myelin antigens, only a fraction of infiltrating CD4^+^ and CD8^+^ T cells are responsive to the immunizing antigen in EAE models (1). Indeed, there has been remarkably limited investigation into CD8^+^ T cell targeting of additional neural epitopes, including neuron-restricted antigens that may be liberated and made accessible during the demyelinating disease process.

We have previously shown that CD8^+^ T cells are both necessary and sufficient to elicit profound axonal injury and loss of motor function within the context of Theiler’s virus-associated demyelination (36, 48–51). Specifically, when purified perforin-competent CD8^+^ T cells isolated from the spinal cords of infected wild-type mice were transferred into profoundly demyelinated but functionally intact perforin-deficient recipient mice, the transferred CD8^+^ T cells rapidly and irreversibly impaired motor function and substantially reduced the number of medium- and large-caliber spinal axons via a viral antigen-independent mechanism (36). We also determined in an *in vitro* model that IFNγ-induced presentation of neuron-restricted antigens on MHC I renders axons vulnerable to perforin- and granzyme B-dependent injury mediated by antigen-specific CD8^+^ T cells (52). These findings unequivocally demonstrated that CD8^+^ T cells—acting through perforin-dependent cytotoxic mechanisms—are sufficient to directly injure demyelinated axons *in vivo* and MHC I + axons in vitro. Furthermore, this dissociation between demyelination and axonal injury in the absence of perforin-mediated CD8 + cytotoxicity provided crucial evidence that demyelination alone is insufficient to cause axonal injury. Conversely, immunodepletion studies in this model confirmed the necessity of CD8^+^ T cells for the maintenance of ongoing axonal injury.

In follow-up studies, we demonstrated that demyelination alone is sufficient to drive neuronal and axonal expression of MHC I molecules loaded with self-antigen, and that demyelination also drives recruitment of CD8^+^ T cells directed against such self-antigens into the brain, resulting in axon injury. Specifically, we found that in the context of neuronally restricted expression of the proto-antigen ovalbumin, cuprizone induced demyelination caused profound recruitment of OVA-specific OT-I effector CD8^+^ T cells to the CNS, and injury of OVA+ axons both *in situ* and ex vivo (21). In line with these previous findings, we have hypothesized that a population of clonally expanded CD8^+^ T cells present in the demyelinated lesions of patients with MS is specific for neuronal antigens and directly injures demyelinated axons, leading to permanent loss of neurologic function and MS disease progression (53). CD8^+^ T cells predominate in active MS lesions, outnumbering CD4^+^ T cells by 10-fold, with oligoclonal expansion patterns distinct from polyclonal CD4^+^ T cell responses (54, 55). Single-cell TCR-*β* sequencing of microscopically isolated cells from demyelinating lesions showed that individual CD8 + clones accounted for up to 35% of all lesional CD8^+^ T cells. Similarly, identical CD8^+^ T cell clones were shown to persist across the brain, cerebrospinal fluid (CSF), and blood compartments for over 5 years, indicating long-lived, antigen-specific responses not observed in CD4^+^ T cell populations (56). There is a strong positive correlation between CD8^+^ T cell frequency and acute axonal injury in MS lesions (57–59), with CD8^+^ T cell number (but not CD4^+^ T cells or total CD3 + T cells) correlating significantly with axonal injury markers. Confocal microscopy has revealed CD8^+^ T cells in close contact with demyelinated axons, with granzyme B-containing cytotoxic granules polarized toward target cells—morphological evidence of active cytotoxic engagement (60) suggesting that these persistent, clonally expanded CD8 + populations represent bona fide disease drivers rather than bystanders. Moreover, the extent of axonal injury correlates robustly with disability and gray matter atrophy (61, 62).

Prior studies of CD8^+^ T cell responses in animal models of MS have been limited by reliance on genetic approaches that lack cell-type specificity, including global CD8^+^ T cell deficiency (63) or global deletion of β2M, which are inherently confounded by the potential regulatory roles of CD8^+^ T cells in both in peripheral immune regulation and within the CNS, as well as developmental effects on the maturation of the immune system. These limitations are compounded by the observation that global β2M deficiency paradoxically worsens EAE severity with enhanced demyelination, axonal damage, and mortality compared to wild-type mice (64), in contrast to studies in TMEV demyelinating disease (23, 24). This effect has been speculated to be due to the abrogation of cytotoxic T cell-mediated killing of other pathogenic lymphocyte populations (58, 65–70). Antibody-mediated CD8^+^ T cell depletion has been employed to circumvent these limitations and provide more acute and reversible approaches to study CD8^+^ T cell contributions to demyelinating disease. These studies revealed that CD8^+^ T cell removal preserves motor function and limits axon loss in chronically demyelinated mice without altering other disease parameters (36).

Here, we show that surface antigen presentation of CNS neuron-restricted antigen is critical for surveillance by the peripheral immune system. We also show that MHC I antigen presentation on neurons contributes to clinical severity and is necessary for neurons to be targeted for elimination by antigen-specific CD8^+^ T cells in the context of cuprizone-induced demyelination. In line with our prior work in TMEV demyelinating disease models, these studies suggest that demyelination is necessary but not sufficient for axonal injury and neurological deficits in mouse models of MS, whereas CD8^+^ T cells are necessary and sufficient for axonal injury and disability in both EAE and cuprizone models of demyelination.

It’s important to note that while deep characterization of CNS-infiltrating CD8^+^ T cells is beyond the scope of the present study, these cells are likely not monolithic in terms of TCR specificity as well as memory, effector, and regulatory subsets. In both AD and MS, clonotyping and single-cell and TCR-sequencing studies have identified clonally expanded CD8 + populations with distinct activation and tissue-homing programs (56, 71, 72), while transcriptomic studies in aged and AD mouse brains support a tissue-resident memory-like phenotype (73, 74). CNS CD8^+^ T cells have been suggested to limit inflammation by targeting other autoreactive lymphocytes or by suppressing microglial activation. In EAE, autoregulatory CD8^+^ T cells can suppress disease by directly targeting encephalitogenic CD4^+^ T cells. Others have identified CXCR6^+^ CD8^+^ T cells that accumulate in the aged brain (75–77). In AD models, CXCR6 + PD-1 + brain-resident CD8^+^ T cells restrain pro-inflammatory microglial cytokine production, and loss of either CXCR6 or CD8^+^ T cells increases microglial inflammatory output and worsens pathology (78, 79) while other AD-associated T cells may promote pathology (80, 81).

While we do not examine the antigen-specificity of CNS-infiltrating CD8^+^ T cells in EAE, others have described CD8^+^ T cells accumulating in the CNS during EAE with specificity for cryptic apoptosis-associated epitopes (82). CD4^+^ T cell responses in MOG EAE have also been shown to exhibit polyreactivity toward neurofilament medium in chronic disease (1), and CD8 crossreactivity toward EBV and neuronal proteins has been suggested in MS (71). Outside of MS, CD8^+^ T cell responses toward neuron-restricted proteins have been described in patients with autoimmune encephalitis and paraneoplastic disease (83–87). Moreover, CD8 T cell specificity for an array of antigens has been suggested to be targeted across neurodegenerative diseases, including *α*-synuclein, Aβ, tau, TDP-43, and mitochondrial components (88–90).

Given that ablating expression of β2M in OVA+ neurons caused reduced activation levels of OT-I T cells in brain-draining deep cervical lymph nodes and profoundly reduced recruitment of these T cells to the CNS in the cuprizone model, it was surprising that the same ablation strategy in EAE did not result in reduced accumulation of CD8^+^ T cells in the brain and spinal cord. However, it’s important to note that neither CD4 nor CD8 T cells are strongly recruited to the CNS in the cuprizone model, in the absence of neuronal expression of the ovalbumin protoantigen and adoptive transfer of ovalbumin-specific T cells. Additionally, we did not test the impact of ovalbumin-specific CD4 T cells (OTII)—which are clearly dispensable for OT-I T cell recruitment in this context but may contribute to broader inflammatory signals that promote accumulation of other patrolling T cells in the CNS. By contrast, both CD4^+^ and CD8^+^ T cells recruited to the CNS in the context of EAE, and notably even the recruited CD8^+^ T cells, are inherently polyclonal, with a range of antigen specificities to both immunizing and non-immunizing antigens. As such, ablating neuronal β2M expression would be unlikely to affect recruitment of either CD4^+^ T cells or CD8^+^ T cells specific for non-neuronal antigens. Moreover, these other (e.g., myelin-reactive) T cells likely contribute to an inflamed blood–brain barrier that is more permissive to T cell infiltration of the CNS. In contrast, T cell recruitment to the CNS is far more limited during cuprizone intoxication—with OVA-specific OT-I T cells likely driving the predominance of lymphocytic CNS infiltrate in our model. Therefore, ablating the capacity of these NAS T cells to effectively recognize their antigen on neurons may have a greater impact on overall T cell recruitment. While further work is needed to determine the extent to which neuronal antigens are targeted by CD8^+^ T cells in patients with MS, our findings suggest that demyelination-driven upregulation of MHC I on neurons contributes to peripheral immune surveillance against neuron-restricted antigens and increases the vulnerability of demyelinated neurons to immunological attack by NAS T cells. Future work should aim to determine the therapeutic value of ablating neuronal MHC I expression in the context of ongoing neuroinflammatory demyelinating disease and the extent to which the presence of CD4^+^ T cells against a shared neuronal antigen promotes neuronal injury even in the context of ablated neuronal MHC I expression.

## Data Availability

The raw data supporting the conclusions of this article will be made available by the authors, without undue reservation.
