# Supplementary material for: Neuronal expression of β2M and MHC I are essential for peripheral surveillance and targeting of neuron-restricted antigens
Source: Front Neurol. 2026 Jun 24;17:1813496. doi: 10.3389/fneur.2026.1813496 (PMC13341288; doi:10.3389/fneur.2026.1813496)
Supplement: Supplementary file 1 [file Data_sheet_1.pdf]

Supplemental Materials

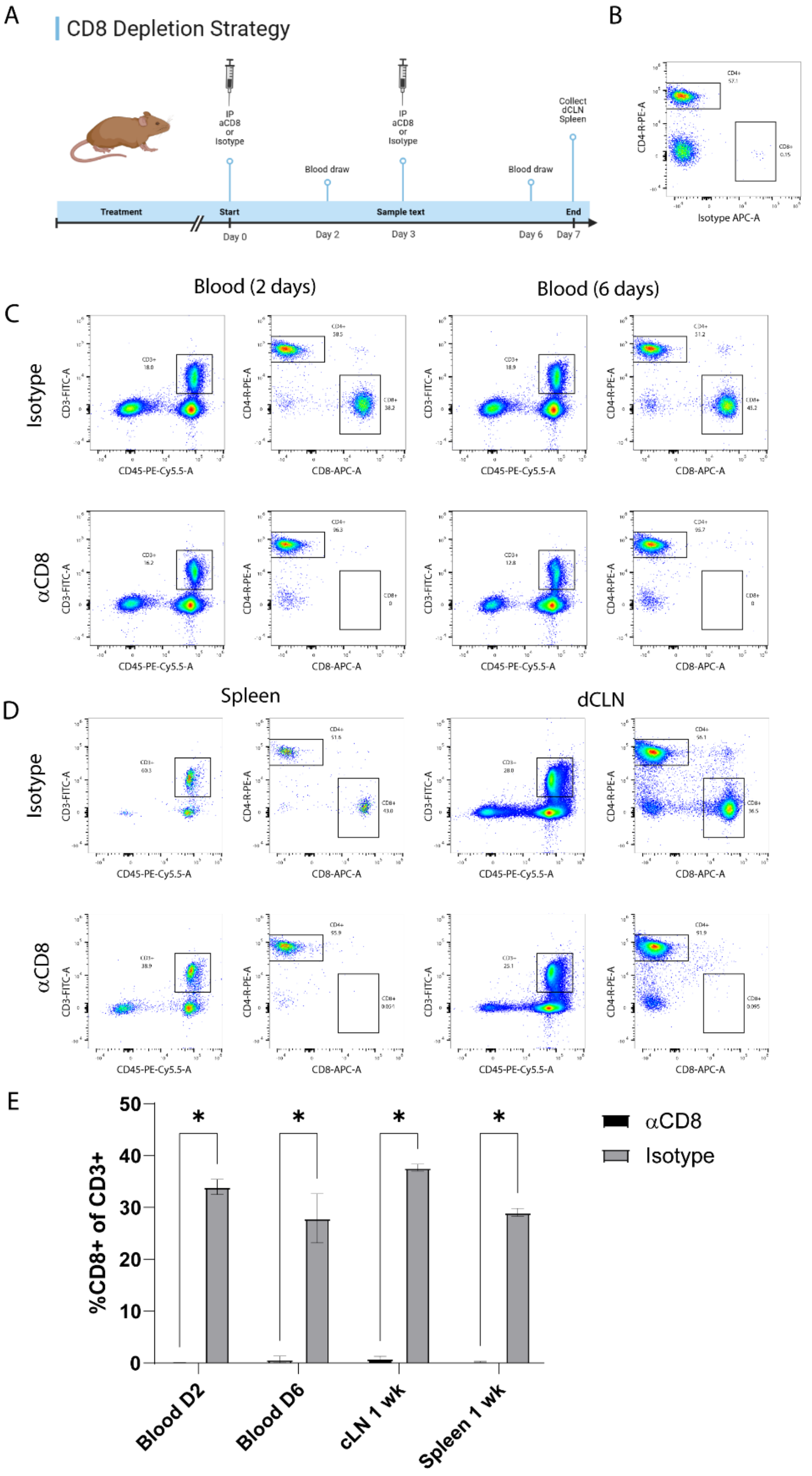

Supp. Figure 1. CD8 depletion strategy and validation by flow cytometry. (A) Schematic of the experimental timeline for CD8 depletion using anti-CD8 or isotype control antibody administration, with blood collection at day 2 and day 6, followed by harvest of cervical lymph nodes (cLN) and spleen at 1 week. (B) Representative flow cytometry plot showing isotype control staining for CD8 (B) as well as CD8 depletion efficiency (C) in peripheral blood at 2 days and 6 days after treatment and (D) in spleen and cervical lymph node at 1 week after treatment in the isotype and anti-CD8 groups. (E) Quantification of CD8+ T cells among CD3+ cells in blood, cLN, and spleen following anti-CD8 or isotype treatment. Data are shown as mean ± SEM. *P < 0.05.

Gating Strategy

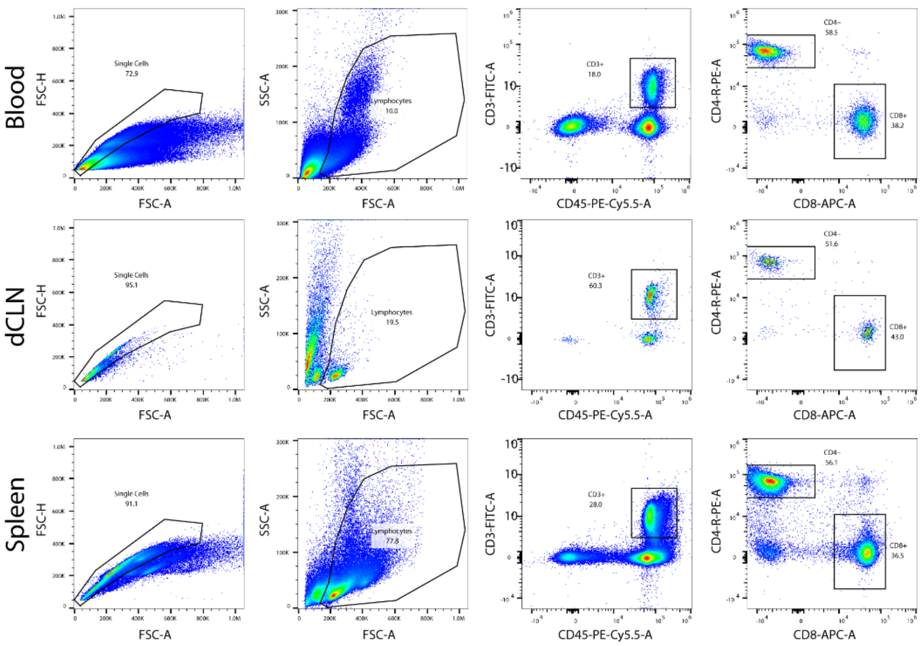

Supp. Figure 2. Flow cytometry gating strategy for immune cell analysis in blood, cervical lymph node, and spleen. Gating strategy used to identify singlets, lymphocytes, CD3+ T cells, and CD4+/CD8+ T-cell subsets. Cells were first gated on forward scatter area/height to exclude doublets, then on lymphocyte FSC/SSC characteristics, followed by CD45+CD3+ T-cell gating and subsequent discrimination of CD4+ and CD8+ populations. Representative plots are shown for blood, deep cervical lymph nodes (dCLN), and spleen.

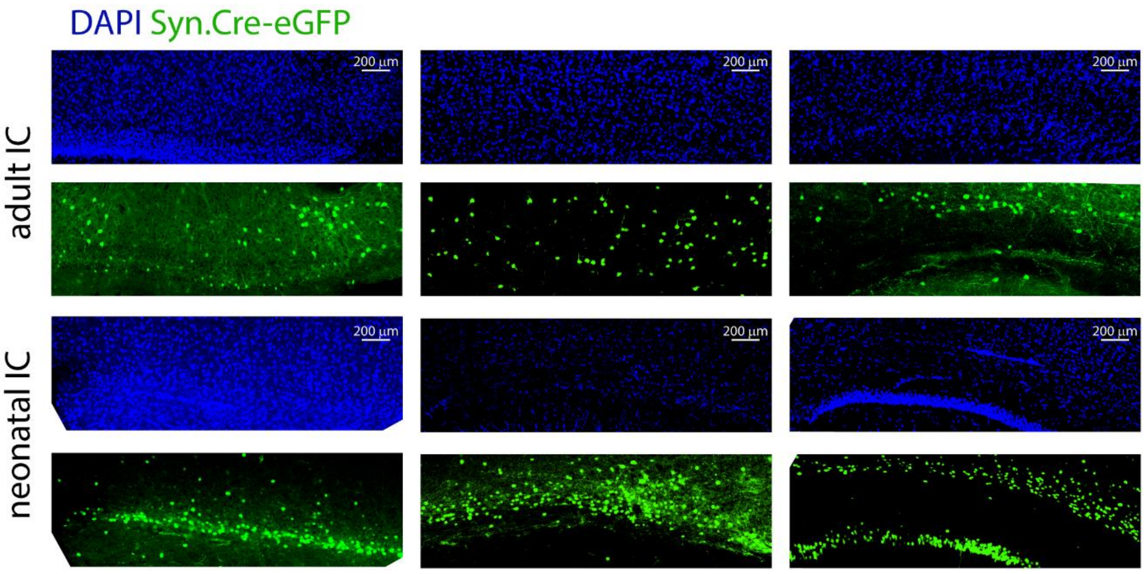

Supp. Figure 3. Representative hippocampal and cortical transduction after AAV1.Syn.Cre-EGFP delivery in neonatal and adult mice. Representative fluorescence images of the hippocampus and overlying cortex from (n = 3) mice injected with AAV1.Syn.Cre-EGFP either at postnatal days 0–2 (neonatal) or at 6–8 weeks of age (adult). DAPI is shown in blue and Syn.Cre-EGFP signal is shown in green. Images illustrate the spatial distribution and relative extent of viral transduction across the injected regions in neonatal versus adult animals. Scale bar, 200 µm.
